# The Use of Non-Cohesive Gold

**Published:** 1906-09

**Authors:** John I. Hart

**Affiliations:** New York, N. Y.


### THE USE OF NON-COHESIVE GOLD.

By Dr. John I. Hart, New York, N. Y.

The properties of gold have been well understood for many years; but few have failed to take advantage of these properties. The placement of non-cohesive gold by the wedge principle is as old as dentistry, and no man who has practiced for many years has failed to observe how fillings of this class have preserved teeth. It is difficult to build out with this material to any considerable extent, and consequently many who desire to restore lost contour have resorted to the use of cohesive gold. I consider that a combination of the two forms of gold facilitates our work and saves fatigue to the patient and strain to the operator. I do not question that the results obtained by those who employ cohesive foil are as satisfactory as I can obtain with the combination material, but I do contend that the effort of the former is much more laborious, and I am insistent that those who work entirely with cylinders cannot obtain as satisfactory results with cohesive cylinders at the gingival seat as can be obtained with non-cohesive cylinders at that situation.

All pure gold is cohesive unless gases have accumulated on the surface, which render it greasy. If gases are allowed to so accumulate the gold partially loses its cohesive property, and, unless annealed, works practically as does non-cohesive foil, but on annealing those gases are driven off, and it becomes fully cohesive.

That is not true of non-cohesive gold; there is only one manufacturer today who prepares absolutely non-cohesive gold, and by that I mean gold that can be annealed to cherry redness, and on being so annealed does not regain cohesive properties.

It is assumed that the method of preparing this foil is that the beaten foil is placed in a furnace between sheets of paper and allowed to remain in the muffle until the paper is incinerated. I say it is supposed that is the process, because it is more or less a secret process; others claim that there is a small percentage of lead with the gold, but in our working with non-cohesive foil we obtain the full advantage of the softness of the pure foil, and non-cohesive foil has been misnamed soft foil, because when worked in cavities in the deeper portions of these cavities it does not stick sheet by sheet and become stiff, and consequently it is called soft foil; but practically at the inception it is no softer than cohesive foil, and in working in deeper portions of the cavity it does not become so stiff.

Tin and gold in combination work precisely as does non-cohesive foil.

Perhaps I can bring to your minds very closely the difference in the working qualities of the two classes of cylinders, if we consider for a moment the difference between stiff electric wire and flexible electric wire. You all know that flexible electric wire is made up of a series of strands of wire interwoven into a small cable. Take, for instance, such a cable of the calibre of my finger, and then take a solid wire of the same calibre, and the difference between the flexibility of the two wires is very marked. That illustrates the difference between cohesive cylinders and non-cohesive cylinders. When a cohesive cylinder is pressed down into the deeper portion of a cavity, each layer of foil making up the cylinder will stick or cohere together, and consequently the series of the sheets which go to make up that cylinder under pressure finally become ten times as thick as the original sheet and as stiff as though it had originally been foil of number thirty if the foil making up the cylinder is composed of number three foil; whereas, if that cylinder is made up of non-cohesive foil it can be pressed down into the corner of the cavity and one sheet of the foil does not stick to the other sheet of the foil; and if it is moved slightly in bringing it down into position, it does not become stiff under the pressure.

My reasoning that the use of non-cohesive cylinders at the gingival seat is more advantageous than the placement of cohesive cylinders at that point, is worked out from this basis: That if the model I have before you is correct, then the retention points made, which are very slight at the angle created by the buccal and gingival walls and by the lingual and gingival walls, cannot be started with cohesive cylinders which may not rock before the filling is completed; but if we wedge in with non-cohesive cylinders we can still prepare cavities on this plan without encroaching on the lateral walls of the teeth and weakening them, or without encroaching on the pulpal wall with damage to the pulp from thermal shock, but by combining with the non-cohesive cylinders, cohesive cylinders, by interdigitation we obtain as firm an anchorage as we would if we had started fresh with the cohesive cylinders in deeper cavities.

A series of cylinders of suitable length are selected, and they are placed on the gingival seat of the cavity with only the pressure than can be brought to bear by foil pliers. Prior to their condensation the series of cohesive cylinders are pressed into place with the same pliers, and then condensation is completed with hand plugger points. After these have been pressed home with the hand plugger points the condensation is completed with a mallet. Then the margins are burnished with a flat smooth burnisher and all the margins are protected by burnishing the excess over the margin. This portion of the filling is now finished with strips. At that stage of the filling, the matrix is put in position and the filling completed with cohesive gold cylinders. The advantage of burnishing and finishing off at the gingival border prior to the completion of the filling is that this is the most difficult portion to finish after the filling is completed. The knuckle or contour of many a filling has been destroyed in the efforts to finish perfectly the gingival margin, when we have waited until the final completion of the filling to finish at that point.

On two occasions recently, in listening to talks on methods of operative procedure, I have heard outlined the difficulties of filling cavities entirely with gold in bicuspids and molars, and the method has been described whereby the operator has completed one-third of the operation of the filling with amalgam and finally completed it with gold; but if that more or less "sloppy" method in the hands of some operators is necessary, then some method is necessary which shall make possible the entire filling with gold of cavities of this description. While I do not offer this as an original method, I suggest it to you because I hope and think it will facilitate your work.—*Items.*

